# Innovation in Aging

**DOI:** 10.1093/geroni/igaf122.1233

**Published:** 2025-12-31

**Authors:** Michelle Putnam

**Affiliations:** University of Massachusetts Boston, Boston, Massachusetts, United States

## Abstract

Innovation in Aging is an interdisciplinary, Open Access journal of the Gerontological Society of America (GSA). Innovation in Aging publishes innovative, conceptually sound, methodologically rigorous research on aging and the life course that has high potential for translating into positive individual, community, and societal outcomes in later life. Submissions are encouraged from a wide range of disciplines including technology, engineering, architecture, economics, business, law, political science and public policy, education, public health, social and psychological sciences, social work, biomedical and health sciences, and the humanities and arts. Innovation in Aging seeks to forward gerontological science by identifying emerging issues, advancing new frameworks for understanding complex problems, presenting cutting-edge methodological approaches and data analysis techniques, and supporting creative thinking about long-standing as well as novel issues and areas of study. The journal also seeks submissions that deepen and broaden understanding of aging, the life course, and later life experiences in new ways. Submissions to Innovation in Aging should articulate what is innovative about the work being presented and how it is translatable and useful for relevant stakeholders and constituencies.

